# Is There a Bacillus Lepræ?

**Published:** 1882-05

**Authors:** 


### IS THERE A BACILLUS LEPRÆ?

Within the last ten or twelve years, several pathologists who had opportunities of examining microscopically the cellular infiltrations found in leprous tubercles have announced the discovery of a peculiar form of bacillus, which was assumed to be the specific cause of leprosy, and in consequence leprosy was forthwith ranked among those diseases supposed to be due to the presence of a parasite or germ. The first observer who announced the discovery of the parasitic organisms in leprous tissues was Armauer Hansen. More recently, Neisser, Carter, Hillairet, Gauché, Bermann, and Cornil all claim to have found the specific organism, which has been labelled the *bacillus lepræ.* In spite of the fact, however, that the observers mentioned insist strenuously on the accuracy of their observations, dermatologists generally have been unwilling to accept their decision as final. In the April number of the *Chicago Medical Journal and Examiner,* Dr. H. D. Schmidt, Pathologist to the Charity Hospital of New Orleans, reviews the evidence for the existence of the bacillus lepræ, in the light of his own observations, and comes to the conclusion that the specific bacillus said to cause leprosy is a myth, and that there is not sufficient evidence to admit the new claimant as a factor in the causation of the disease. Dr. Schmidt prepared sections of leprous tissue according to the directions of Dr. I. Bermann, of Baltimore, one of the most recent of these observers, with the following results. He says:

"In subjecting these sections to a close microscopical examination, I failed, notwithstanding their remarkable thinness and transparency, to detect even a single bacillus. The only parasitic organisms met with were an inconsiderable number of patches, or so-called zoöglœa of micrococcus, thoroughly stained by the bright blue of the methyl violet; but these, even, were principally confined to the sections of skin taken from the lobules of the ear from the first and third cases. The most of these patches were small, and ellipsoidal, or spindle-shaped in form, the smallest corresponding in size and outline to the larger or smaller spindle-shaped cells met in the corium of the skin, while the larger ones were generally composed of a number of the former, either placed one behind the other, or, in some instances, overlapping one another. In most instances, the long axis of these ellipsoidal, or spindle-shaped zoöglœa, was placed parallel with the surface of the skin, or with the course of the connective tissue bundles of the corium, though others were observed placed obliquely, or, also, to present irregular outlines. A few larger patches, round or oval in outline, and corresponding to the shape of a transverse or oblique section of a larger vessel, were likewise met with. In all these patches of micrococcus, however, the individual micrococci could be distinctly recognized, particularly by their bright blue color, contrasting with the very feeble picro-carmine staining of the sections ; the original structure of the latter showed as perfectly as fifteen months before, when the sections were cut. In the skin taken from the second case, no trace of micrococcus, even, could be detected.

"But, as regards the micrococcus-zoöglœa observed upon the sections of skin, it must be remarked that they were only found upon the corium of the skin on one side of the lobule, the other side being perfectly free, and, by alternately changing the focus, it was furthermore found that the patches of micrococci rested almost always upon one or the other surface of the section ; in a few exceptional instances, only, the minute patch appeared to pass into the section. But even in these instances a closer examination revealed them deposited in some interspace of the connective tissue bundles, or in a vessel laid open by being cut in an oblique or longitudinal direction. The ellipsoidal or spindle-shaped form, which, perhaps, the greater part of these patches or zoöglœa presented, was very probably caused by the settling of the first individual micrococci upon one of those numerous spindle-shaped cells, met with in the corium, or upon a connective tissue bundle very obliquely cut, in consequence of which the patch, arising from those first settlers, finally assumed the form of the host upon which it grew. In the same manner, the colony may, in other instances, have assumed the form of a vessel by having settled and grown into the lumen laid open by the knife. Those zoöglœa of a round or oval form, generally larger in size, and by their greater thickness less transparent, were most probably deposited in the transversely or obliquely cut lumen of a large vessel. Now, whether the ellipsoidal or spindle-shaped form of the patches were really determined by the causes I have pointed out above, or whether the latter assumed these forms independently of these circumstances, I would not positively assert, though the explanation I have offered appears to be the most plausible. Neither do I deem it necessary to further speculate, in this place, as to the cause of the irregular forms, or those patches placed obliquely to the course of the fibrous bundles of corium ; though, before dismissing the skin, I repeat : *that in no instance did I ever observe micrococci lodged in the interior of any of the cells*, notwithstanding the sections being sufficiently thin to have shown the parasites, even without staining."

"Finally, the exceedingly fine filaments of a fungus, about $\frac{1}{1600}$ m.m. in thickness remain to be mentioned ; they were observed to spread *over* some sections of tongue, liver, kidney, and lymphatic gland, but in no instance entered the tissue ; on the contrary, in order to render these filaments distinct, the objective had to be adjusted so far away from the section as to bring the histological elements of the latter entirely out of focus.

"It appears to me almost superfluous to enlarge to any extent upon the signification of the presence of the micrococcus-zoöglœa, or upon that of the fine fungous filaments, found, as stated above, upon some of the sections, for it is obvious that they are deposited after the latter were cut. And, in regard to the fungous filaments, I hesitate not to say that, being very readily stained by hæmatoxylin, I have observed them well enough during my first examinations in the pathology of the disease—but, knowing, at that time, that they bore no relation whatever to the tissues I was then studying, it never entered my mind to create a sensation by mentioning them in my paper. At what particular period of the process of preparing the sections they found their way upon the latter, I am unable to say, but it must be known to every practical histologist that solutions of carmine, or of hæmatoxylin, do frequently attract fungi, and that, in the above case, the germs may have settled upon the sections while lying in the staining fluid, or, subsequently, in the mixture of alcohol and water, in which I generally preserve my sections before they are wanted. The micrococci, found upon the sections which I recently examined, may have been deposited since the time of my first examination—that is, while they were kept—each kind of section in a separate bottle—in the alcoholic solution. This becomes the more probable in calling to mind the sections of skin, upon which the micrococci colonies were only found deposited upon one half of the surface, for the reason that the other half was very likely protected by another section lying upon it.

"But let us return once more to the bacilli met with in the leprous tissues by the above-mentioned observers, and, while admitting the truth of their statements, ask for the signification of the phenomenon, or its bearing upon the origin of leprosy. There is no other answer to this question, but a simple denial of any relationship existing between these organisms and the original cause of leprosy, until they can be shown in the blood of *every case* of this disease, and, more, *in such numbers* as to render their noxious influence upon the human organism evident. We may furthermore ask an explanation for the total absence of bacilli in the numerous sections—taken from the organs of three typical cases of leprosy—which I examined, even with the assistance of the bacteria-detective, the methylviolet. The discrepancy existing between the results I obtained myself and the statements of the above observers, can only be explained by either presuming that some mistake has been made on one side or the other, or by supposing the bacilli to have entered the tissues from without. The latter may take place through an ulcerated tubercle, or by the deposition of some of the organisms upon the sections in the manner I have explained above ; or, also, the bacilli may have found access into the pieces of tissue after these were removed from the cadaver and before the cutting of the sections ; this supposition is applicable to the tissues Neisser had before him. There are a number of ways by which bacilli may enter the tissues, leaving aside the possibility of these organisms arising as products of the disease in the living tissues themselves, a view which does not lack supporters. At any rate, the tissues which I examined were tolerably fresh, the autopsies having been made but a few hours after death.

"In conclusion, I may say that the opposition, taken by me in the above review against the theory that leprosy is caused by minute parasitic organisms, is not based upon preconceived notions ; on the contrary, I am far from denying the possibility of bacilli causing certain diseases, as, for instance, splenic fever, though here, it must be remembered, the bacilli are found in the blood itself, or to obstruct the smaller blood-vessels and lymphatics in the form of emboli, while, as far as I know, they have never been observed in the protoplasm of cells. But admitting, even, that such a phenomenon actually presented itself in leprosy, its explanation, that is, the way by which these organisms entered the interior of the cells, would still remain obscure unless we take recourse to the supposition that they originated in the protoplasm itself. Excluding, however, their origin within the tissues, there remain only two ways by which they could enter the human organism ; these are, either through an open lesion, or by entering the blood through the lungs or alimentary canal ; and, it is not at all improbable that in some instances they may enter through an open lesion, especially at the time when the tubercles commence to undergo softening and ulceration, such as Hansen has stated. Their presence in the blood this author has repeatedly denied. In my own examinations I have never observed the slightest trace of a bacillus in the smaller blood-vessels, notwithstanding the sections examined were sufficiently thin for recognizing with the greatest distinctness the blood-corpuscles contained within. Besides, the clinical history of leprosy is so well known that I may refrain from further showing the difficulty of making the characteristic symptoms of this disease harmonize with the bacteria theory ; in every instance where bacteria or bacilli are really met with in the tissue it must be presumed that they arrived there by accident and in search of a nutritive soil for their development. But as it is desirable that the truth should be ascertained, and as I fortunately still have numerous sections of various leprous tissues—kept in alcohol and water—on hand, I shall be pleased to furnish, upon application, a number of them to any competent pathologist, who would like to investigate the subject with the view of publishing the results of his examinations.''

